# External Stenting for Saphenous Vein Grafts in Coronary Surgery: A Systematic Review and Meta-Analysis

**DOI:** 10.3390/jcm12237395

**Published:** 2023-11-29

**Authors:** Giovanni Jr Soletti, Arnaldo Dimagli, Lamia Harik, Gianmarco Cancelli, Roberto Perezgrovas-Olaria, Talal Alzghari, Michele Dell’Aquila, Jordan Leith, Sabrina Castagnini, Christopher Lau, Leonard N. Girardi, Mario Gaudino

**Affiliations:** 1 Department of Cardiothoracic Surgery, Weill Cornell Medicine, New York, NY 10065, USAtja2001@med.cornell.edu (T.A.);; 2 Department of Cardiac Surgery, Sant’Orsola-Malpighi Hospital, 40138 Bologna, Italy

**Keywords:** coronary artery disease, CABG, saphenous vein graft, VEST, external stent, graft occlusion, intimal hyperplasia, repeat revascularization

## Abstract

The external stenting of saphenous vein grafts (SVGs) during coronary artery bypass grafting (CABG) has been proven to reduce intimal hyperplasia (IH) in animal models, paving the way for human randomized controlled trials (RCTs) to be conducted. Herein, we performed a study-level meta-analysis to assess the impact of the Venous External SupporT (VEST) device, an external stent, on the outcomes of SVGs. A systematic search was conducted to identify all RCTs comparing VEST-stented to non-stented SVGs in patients undergoing CABG. The primary outcome was graft occlusion. The main secondary outcomes were repeat revascularization, SVG IH area, and intimal-medial thickness. Two RCTs totaling 407 patients were included. At a mean follow-up of 1.5 years, there was no difference in graft occlusion between groups (incidence rate ratio: 1.11; 95% confidence interval (CI): 0.80–1.53). The rate of repeat revascularization was also similar (odds ratio: 0.66; 95% CI: 0.27–1.64). The IH area (standardized mean difference (SMD): −0.45; 95% CI: −0.79 to −0.10) and intimal-medial thickness (SMD: −0.50; 95% CI: −0.90 to −0.10) were significantly reduced in the VEST group. Our findings show that significant reductions in the IH area and the intimal-medial thickness in VEST-stented SVGs do not currently translate into a lesser need for repeat revascularization or less graft occlusion events compared to non-stented SVGs at 1.5 years after CABG.

## 1. Introduction

Despite the rising adoption of multiple arterial grafting strategies, saphenous vein grafts (SVGs) continue to be the most used conduits in coronary artery bypass grafting (CABG) [1]. Their widespread utilization is mainly due to the fact that SVGs are easily harvested and technically simple to use because of their wall characteristics and larger diameter compared to arteries. Furthermore, because of their length, SVGs can reach any target of the coronary artery system and can be even used to graft multiple vessels. However, their long-term durability is suboptimal, with up to 40% being occluded 10 years after CABG [2]. The key drivers of SVG failure include intimal hyperplasia and the development of graft atherosclerosis [3]. Pharmacological attempts to mitigate intimal hyperplasia, generally, have been unsuccessful [4]. The Venous External SupporT (VEST) device (Vascular Graft Solutions, Israel), an external support for SVGs, has been shown to reduce intimal hyperplasia in pre-clinical models, with the potential to improve long-term SVG patency [5]. This finding has paved the way for randomized controlled trials (RCTs) to be conducted on humans. Herein, we performed a study-level meta-analysis of RCTs that tested the VEST device.

## 2. Materials and Methods

This study conforms to the PRISMA (Preferred Reporting Items for Systematic Reviews and Meta-Analyses) guidelines for transparent reporting [6]. Ethical approval was waived as data were solely collected from published works in the literature.

### 2.1. Protocol Registration

This meta-analysis was registered with PROSPERO, an international database of prospectively registered systematic reviews. Its identification number is CRD42023434757.

### 2.2. Device Description

The VEST device (Vascular Graft Solutions, Tel Aviv, Israel) is an external cobalt-chrome tubular support for SVGs during CABG with a braided structure composed of plastically deformable wires that can be shaped to the length and width of a vein graft. As two models were being produced at the time of our searches, we directly consulted a company delegate who confirmed that all completed RCTs used the same model (VEST 1.0).

### 2.3. Search Strategy

The full search strategy is available in Supplemental Table S1. On 31 January 2023, after summarizing the research question using the PICO (Population, Intervention, Comparator/s, Outcome/s) format, a trained evidence synthesis librarian performed a comprehensive systematic search on three different databases: Ovid MEDLINE (1946 to present), Ovid Embase (1974 to present), and the Cochrane Library. The objective was to identify all RCTs comparing VEST-stented to non-stented SVGs in patients undergoing CABG. No language or publication date restrictions were applied. All non-randomized evidence was excluded.

### 2.4. Study Selection

After identification of the studies, duplicates were removed. Titles and abstracts were then reviewed against inclusion and exclusion criteria by two independent authors (G.C. and S.C.). Remaining reports were subsequently assessed for eligibility, while reference lists were screened for potential studies not included in the initial search.

### 2.5. Data Abstraction and Risk of Bias Assessment

Data extraction was independently performed by two authors (M.D.A. and J.L.). Discrepancies were resolved by the first and senior authors (G.J.S. and M.G.). The following variables were extracted: study characteristics and baseline, intraoperative data, and postoperative data.

The quality of the included RCTs was assessed using the revised Cochrane Risk of Bias tool 2 [7]. Traffic light and summary plots were generated using the “robvis” visualization tool [8].

### 2.6. Outcomes and Definitions

The primary outcome was graft occlusion. Secondary outcomes were repeat revascularization, intimal hyperplasia area, intimal-medial thickness, and lumen irregularities expressed using the Fitzgibbon classification (class I: perfect patency or no lumen irregularities; class II: lumen irregularities that involve less than 50% of SVG length; class III: lumen irregularities that involve more than 50% of SVG length). For the purpose of this analysis, Fitzgibbon classes II and III were considered together.

As in the included trials, the intimal hyperplasia area was defined as the external elastic media area minus the lumen area (or plaque area plus media area), whereas the intimal-medial thickness was defined as the external elastic media diameter minus the lumen diameter (or plaque diameter + media diameter). The intimal hyperplasia area and intimal-medial thickness were assessed via an intravascular ultrasound by advancing a catheter all the way through each study graft (supported and unsupported) and then pulling it back at a constant rate to obtain quantitative measurements. Repeat revascularization included ischemia- and non-ischemia-driven reinterventions of supported or unsupported grafts or associated target coronary artery.

### 2.7. Statistical Analysis

Categorical outcomes were extracted as raw numbers, while continuous outcomes were extracted as mean and standard deviation.

Considering the different lengths of follow-up between the studies, the incidence of follow-up outcomes was linearized over time by, respectively, calculating the incidence rates in the stented and non-stented groups. For each study, the ratio of the incidence rate (IRR) was calculated, and IRRs were then pooled meta-analytically using the inverse variance method.

Similarly, standardized mean difference (SMD) was calculated for continuous outcomes from each study and pooled meta-analytically using the inverse variance method.

Random-effects models were used. *I*^2^ was used to describe the proportion of total variability in treatment effect due to between-study heterogeneity. Low, moderate, and high heterogeneities were defined as *I*^2^ < 25%, 25–50%, and >50%, respectively.

A secondary analysis for the primary outcome including the venous external support trial (VEST) IV trial (which was excluded from the main analysis, as detailed in the Results section) was performed. No publication bias or small-study effect analyses were performed since only 2 RCTs were included.

In all analyses, the reference was the non-stented group. A *p*-value of <0.05 was considered statistically significant. Statistical analyses were performed in R version 4.2.3 (R Foundation for Statistical Computing) using the following packages: “meta” and “dmetar”.

## 3. Results

**Figure 1:**
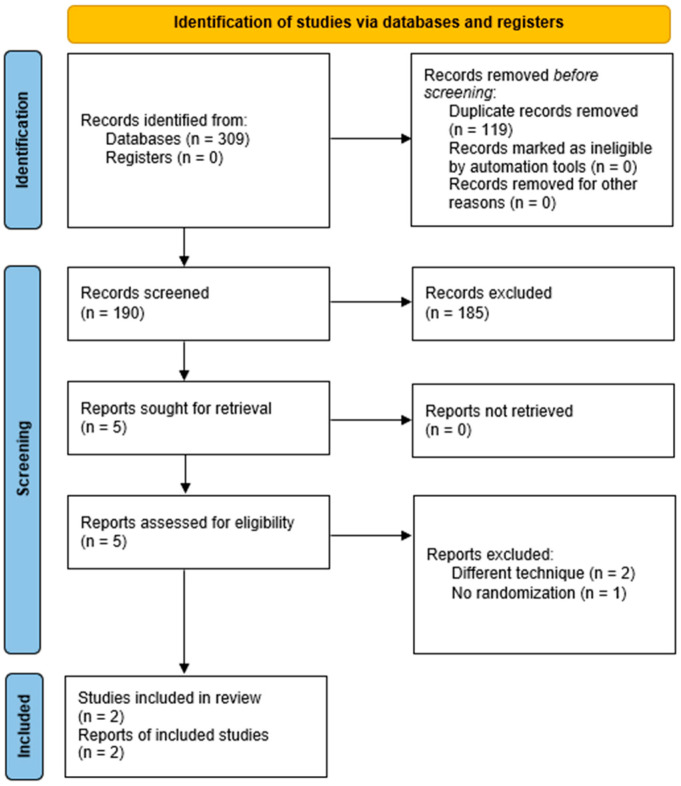
PRISMA flow diagram. PRISMA: Preferred Reporting Items for Systematic Reviews and Meta-Analyses.

The PRISMA flow diagram outlining the study selection process is shown in Figure 1. The included studies were VEST III and US VEST [9,10]. The excluded studies were VEST I, II, and IV. VEST I and its long-term follow-up, VEST IV, were excluded because 30% of the enrolled sample size underwent device implantation with a modified surgical technique which required its fixation to the proximal or distal anastomoses, or both [11,12]. VEST II was excluded because VEST-SVGs were assigned to the right coronary territory, while non-VEST SVGs were assigned to the left coronary territory, and no randomization was performed [13].

The risk of bias assessment is displayed in Supplemental Figure S1A,B. Overall, the assessment showed concerns arising from the randomization process domain for VEST III and showed bias due to missing outcome data for both VEST III and US VEST.

The study characteristics and baseline, intraoperative, and postoperative data are presented in Supplemental Table S2. A total of 407 patients were analyzed. The median follow-up was 1.5 years. The mean age was 66.2 ± 8.1 years. The mean proportion of women was 18.6%. Hypertension (89.1%), hyperlipidemia (86.5%), and diabetes (41.3%) were the most frequent comorbidities. Most patients (55.5%) underwent endoscopic SVG harvesting. The total number of SVGs deployed was 813. Of these, 366 (183 stented SVGs and 183 controls) and 447 (223 stented SVGs and 224 controls) were used in VEST III and US VEST, respectively.

### 3.1. Primary Outcome

**Figure 2:**
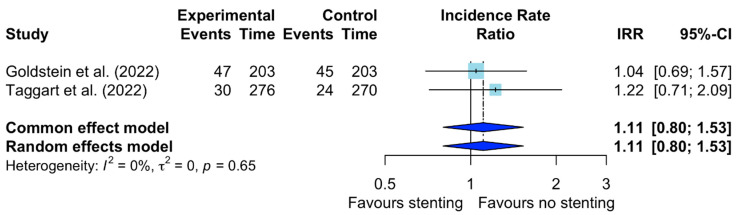
Forest plot for graft occlusion. CI: confidence interval; IRR: incident rate ratio [9,10].

There was no difference in the incidence of graft occlusion between the VEST-stented and non-stented SVGs (IRR: 1.11; 95% confidence interval (CI): 0.80–1.53: *I*^2^ = 0%) (Figure 2). A secondary analysis including VEST IV confirmed this finding (IRR: 1.15; 95% CI: 0.84–1.56; *I*^2^ = 0%) (Supplemental Figure S2).

### 3.2. Secondary Outcomes

**Figure 3:**
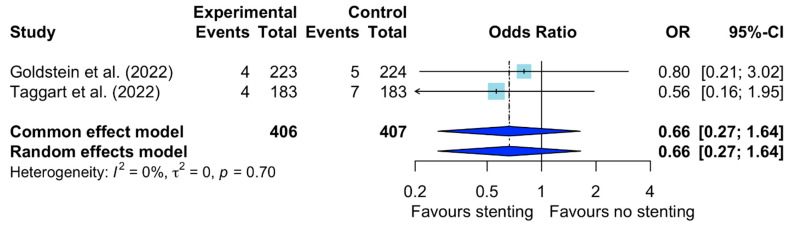
Forest plot for repeat revascularization. CI: confidence interval; OR: odds ratio [9,10].

There was no difference in the incidence of repeat revascularization between the VEST-stented and non-stented SVGs (OR: 0.66; 95% CI: 0.27–1.64; *I*^2^ = 0%) (Figure 3). Additionally, no significant difference between the groups was observed in terms of lumen irregularities expressed as Fitzgibbon class patency I (IRR: 1.15; 95% CI: 0.92–1.44; *I*^2^ = 0%) (Supplemental Figure S3) and Fitzgibbon patency classes II/III (IRR: 0.82; 95% CI: 0.63–1.07; *I*^2^ = 0%) (Supplemental Figure S4). Significant reductions in the intimal hyperplasia area (SMD: −0.45; 95% CI: −0.79 to −0.10; *I*^2^ = 55%) (Supplemental Figure S5) and intimal-medial thickness (SMD: −0.50; 95% CI: −0.90 to −0.10; *I*^2^ = 66%) (Supplemental Figure S6) were found in the VEST-stented SVGs compared to the non-stented SVGs.

## 4. Discussion

In this meta-analysis of two RCTs including 407 patients, we found no difference in terms of graft occlusion between the VEST-stented and non-stented SVGs 1.5 years after CABG. Although the VEST device significantly reduced the intimal hyperplasia area and intimal-medial thickness (atherosclerosis-predicting factors), it did not yield to a lesser need for repeat revascularization.

Diffuse intimal hyperplasia is the late consequence of a primary endothelial injury which mainly results from the excessive dilation of SVGs after exposure to arterial pressure (platelet adhesion and mitogenic protein release are also involved in intimal proliferation processes) [14]. The VEST device is an external support for SVGs that specifically targets altered flow patterns and high wall tension, which are the underlying mechanisms of vein graft disease. With its arterial biomechanical features, VEST has been shown to prevent SVG non-uniform dilatation, mitigating the formation of abnormal flow patterns and the subsequent development of intimal hyperplasia. Theoretically, while this should result in improved hemodynamics, reduced thrombus formation, decreased graft occlusion events, and a lesser need for repeat revascularization, our findings showed that VEST currently does not have an impact on the latter two outcomes. The most logical explanation for this discrepancy is that the mean follow-up of our analysis is only 1.5 years, and a longer follow-up would, in fact, unveil a statistical difference between the groups in terms of graft occlusion and reintervention.

Our results are consistent with a recent meta-analysis which showed that, although the intimal hyperplasia area (SMD: −0.77 mm^2^; 95% CI: −1.10 to −0.45; *I*^2^ = 0%) and intimal-medial thickness (SMD: −0.06 mm^2^; 95% CI: −0.08 to −0.04; *I*^2^ = 0%) were significantly lower in the VEST group, graft failure rates were similar in the two groups (OR: 1.22; 95% CI: 0.88–1.71; *I*^2^ = 0%). Of note, the aforementioned meta-analysis was limited by (1) the inclusion of VEST I, which is known to have adopted a variation of the device implantation technique in 30% of the sample size, and (2) the exclusion of repeat revascularization, a key clinical outcome, from the quantitative analysis [15].

To corroborate such findings, a secondary analysis of US VEST found that larger areas of intimal hyperplasia were associated with worse clinical events at 3 years [16]. However, the clinical implications of this surrogate outcome are unclear, and more specific endpoints (such as repeat revascularization and major adverse cardiac and cerebrovascular events) should be used to demonstrate a substantial clinical advantage of the VEST device over the standard of care [17].

It must also be noted that the SVG occlusion rate in the US VEST was 22.7% (92/406), nearly 75% higher than the anticipated rate (13%) described in the protocol [10]. This higher-than-expected occlusion rate led to an unconventional situation where the primary outcome results were mostly based on imputation techniques (where missing data are replaced with a substitute value to retain most of the information in a dataset), an important limitation. Eventually, the trial failed to show a difference in the intimal hyperplasia area (primary outcome) at 1 year between the groups but, considering its limitations and the findings of its 3-year post hoc analysis, further investigation on long-term graft patency and clinical outcomes is needed.

In this regard, the ongoing VEST EU (EUropean) registry, which is collecting data on ischemia-driven revascularization of VEST-supported SVGs or target coronary artery at 5 years after CABG in patients who will receive at least one stented and one non-stented SVG, will provide further (although non-randomized) critical information.

## 5. Conclusions

In conclusion, our data showed for the first time that the VEST device reduced both the intimal hyperplasia area and the intimal-medial thickness of SVGs after CABG. However, this reduction did not translate clinically into fewer graft occlusions or a lesser indication to repeat revascularization compared to non-stented SVGs at 1.5 years of follow-up. Long-term data and larger studies are needed to clarify the effect of the VEST device on CABG patients. Until then, either SVGs harvested with the no-touch technique or arterial conduits, when clinically indicated, may be better suited to provide long-term graft patency in patients undergoing CABG [18,19].

### Limitations

This study should be interpreted in the context of its limitations. First, we only included two RCTs (only three were available in the literature) with a relatively small sample size and short-term follow-up. Second, the primary outcomes used in the selected RCTs are surrogate endpoints, which are not significant clinical events.

## Data Availability

The data will be available upon reasonable request.

## Supplementary Materials

The following supporting information can be downloaded at: https://www.mdpi.com/article/10.3390/jcm12237395/s1, Table S1. Complete search strategy. Table S2. Study characteristics and patient data. Figure S1. Traffic-light and summary plots for RCT quality assessment using the revised Cochrane risk-of-bias tool 2. Figure S2. Secondary analysis for the primary outcome of graft occlusion including VEST IV. Figure S3. Forest plot for Fitzgibbon patency class I. Figure S4. Forest plot for Fitzgibbon patency classes II–III. Figure S5. Forest plot for intimal hyperplasia area. Figure S6. Forest plot for intimal-medial thickness.
