# Supplementary material for: External Stenting for Saphenous Vein Grafts in Coronary Surgery: A Systematic Review and Meta-Analysis
Source: J Clin Med. 2023 Nov 29;12(23):7395. doi: 10.3390/jcm12237395 (PMC10707268; doi:10.3390/jcm12237395)
Supplement: Supplementary file 1 [file jcm-12-07395-s001.zip › jcm-2557061-supplementary.pdf]

SUPPLEMENTAL MATERIAL

TABLE OF CONTENTS

Supplemental Table 1. Complete search strategy 2

Supplemental Table 2. Study characteristics and patient data 3

Supplemental Figure 1. Traffic-light and summary plots for RCT quality assessment using the revised Cochrane risk-of-bias tool 2 5

Supplemental Figure 2. Secondary analysis for the primary outcome of graft occlusion including VEST IV 6

Supplemental Figure 3. Forest plot for Fitzgibbon patency class I 7

Supplemental Figure 4. Forest plot for Fitzgibbon patency classes II-III 8

Supplemental Figure 5. Forest plot for intimal hyperplasia area 9

Supplemental Figure 6. Forest plot for intimal-medial thickness 10

Supplemental Table 1. Complete search strategy

Ovid MEDLINE (ALL – 1946 to present)

Searched on January 31, 2023

No language, article type, or publication date restrictions

Line #	Search
1	Stents/ and external.tw.
2	(external adj4 (stent or stents or stenting or stented or support or supports)).tw.
3	1 or 2
4	Saphenous Vein/
5	(Saphenous or SVG or saphena vein or saphenous venos system or vena saphena).tw.
6	4 or 5
7	3 and 6

Ovid Embase (1974 to present)

Searched on January 31, 2023

No language, article type, or publication date restrictions

Line #	Search
1	Stents/ and external.tw.
2	(external adj4 (stent or stents or stenting or stented or support or supports)).tw.
3	1 or 2
4	Saphenous Vein/
5	(Saphenous or SVG or saphena vein or saphenous venos system or vena saphena).tw.
6	4 or 5
7	3 and 6

Cochrane Library (Wiley)
Searched on January 31, 2023
No language, article type, or publication date restrictions

ID	Search
#1	(external NEAR/4 (stent or stents or stenting or stented or support or supports)):ti,ab
#2	(Saphenous or SVG or saphena vein or saphenous venos system or vena saphena):ti,ab
#3	#1 AND #2

Supplemental Table 2. Study characteristics and patient data

Trial	VEST III	US VEST	Total
**Year of publication**	2022	2022	-
**Country**	Multicenter (Europe)	Multicenter (North America)	-
**Study period**	October 2015 May 2019	January 2018 February 2019	-
**Follow-up (years)**	2	1	1.5
**Sample size**	183	224	407
**Mean age (years)**	66.6±7.9	65.8±8.3	66.2±8.1
**Female sex**	29/183 (15.8)	46/224 (20.5)	75/407 (18.4)
**Smoking habit***	118/183 (64.5)	123/224 (54.9)	241/407 (59.2)
**Diabetes**	54/183 (29.5)	114/224 (50.9)	168/407 (41.3)
**Mean BMI**	28.6±4.4	30.2±5.6	29.4±5
**Hypertension**	168/183 (91.8)	191/224 (85.3)	359/407 (88.2)
**Hyperlipidemia**	164/183 (89.6)	188/224 (83.9)	352/407 (86.5)
**COPD**	23/183 (12.6)	16/224 (7.1)	39 (9.6)
**Vein varicosity**			
• **None**	146/183 (79.8)	154/223 (69.1%)	300/406 (73.9)
• **Mild/moderate**	37/183 (20.2)	69/223 (30.9)	106/406 (26.1)
**NYHA class ≥ III**	59/183 (32.2)	26/215 (12.1%)	85/407 (21.3)
**Prior MI**	58/183 (31.7)	93/224 (41.5%)	151/407 (37.1)
**Prior PCI**	48/183 (26.2)	55/224 (24.6%)	103/407 (25.3)
**Mean surgery time (min)**	237.4±51.4	268.5±68.5	252.9±59.9
**Mean XC time (min)**	60.4±21.1	83±25.8	71.7±23.4
**Mean CPB time (min)**	97.7±26.8	110.5±35.1	104.1±30.9
**SVG harvesting method**			
• **Open**	118/183 (64.5)	41/224 (18.3)	159/407 (39.1)
• **Endoscopic**	57/183 (31.1)	169/224 (75.4)	226/407 (55.5)
• **Other**	8/183 (4.4)	14/224 (6.3)	22/407 (5.4)

Trial	VEST III	US VEST	Total
**N° grafts per patient**			
• **3**	138/183 (75.4)	137/224 (61.2)	275/407 (67.6)
• **4**	42/183 (22.9)	72/224 (32.1)	114/407 (28.0)
• **5**	3/183 (1.7)	14/224 (6.3)	17/407 (4.2)
• **6**	0/183 (0)	0/224 (0)	0/407 (0)
• **7**	0/183 (0)	1/224 (0.4)	1/407 (0.2)
**N° SVGs deployed**	366 (100)	447 (100)	813 (100)
• **VEST-SVGs**	183/366 (50)	223/447 (50)	406/813 (50)
• **Control**	183/366 (50)	224/447 (50)	407/813 (50)
**N° of patients with angiographic follow-up**	128/183 (69.9)	203/224 (90.6)	331/407 (81.3)
**N° SVGs analyzed**	273/366 (74.6)	406/447 (90.8)	679/813 (83.5)
• **VEST-SVGs**	138/273 (50.5)	203/406 (50)	341/679 (50.2)
• **Control**	135/273 (49.5)	203/406 (50)	338/679 (49.8)
**Graft occlusion**	54/273 (19.8)	92/406 (22.7)	146 (21.5)
• **VEST-SVGs**	30/54 (55.5)	47/92 (51.1)	77 (52.7)
• **Control**	24/54 (45.5)	45/92 (48.9)	69 (47.3)
**Repeat revascularization**	11/183 (6.0)	9/224 (4.0)	20/407 (4.9)
• **VEST-SVGs**	4/11 (36.4)	4/9 (44.4)	8/20 (40.0)
• **Control**	7/11 (63.6)	5/9 (55.6)	12/20 (60.0)

*Current and former. Numbers are presented as counts (percentages) or mean±standard deviation. BMI: body mass index; CPB: cardiopulmonary bypass; COPD: chronic obstructive pulmonary disease; MI: myocardial infection; NYHA: New York Heart Association; PCI: percutaneous coronary intervention; SVG: saphenous vein graft; VEST: venous external support trial; XC: cross-clamp.

Supplemental Figure 1. Traffic-light (**A**) and summary plots (**B**) for RCT quality assessment using the revised Cochrane risk-of-bias tool 2

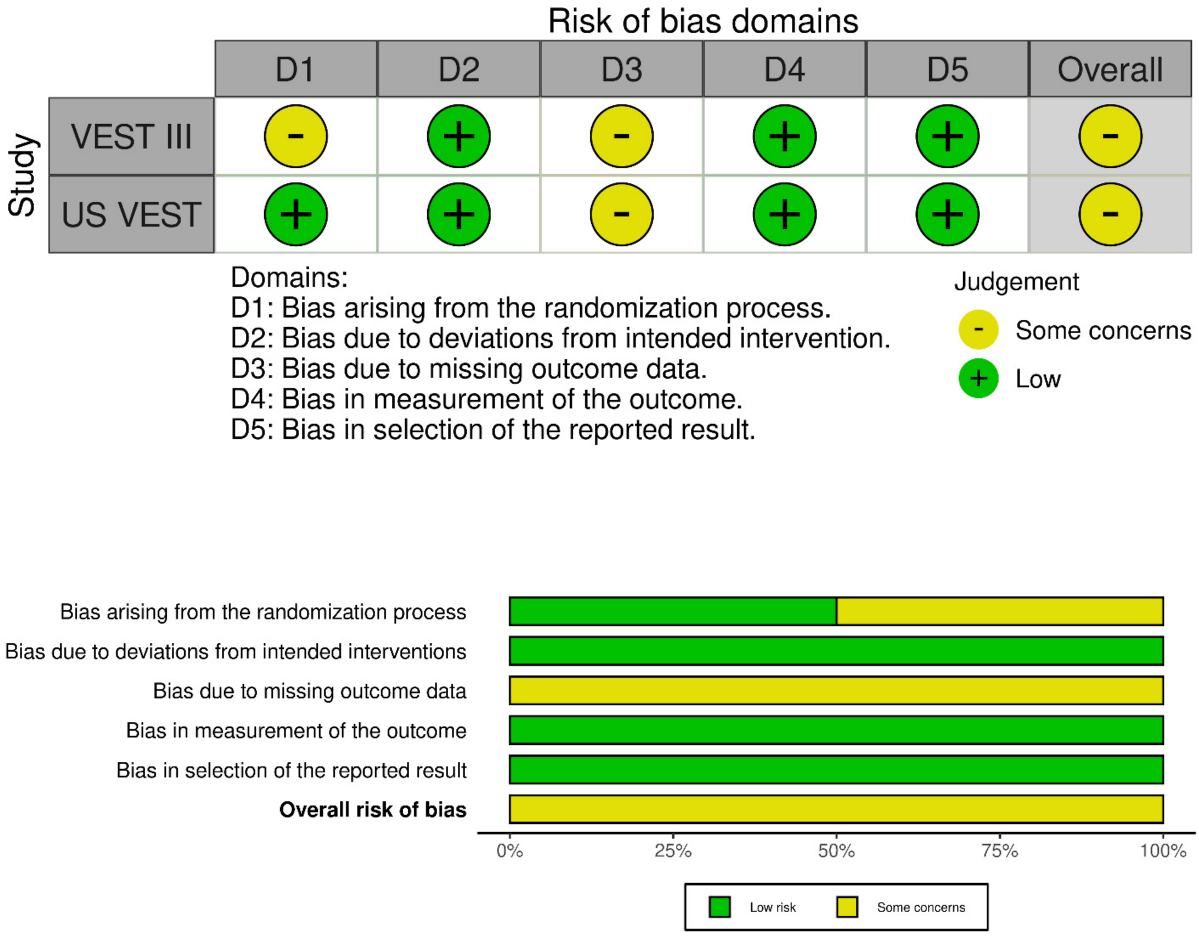

VEST: venous external support trial.

Supplemental Figure 2. Secondary analysis for the primary outcome of graft occlusion including VEST IV*

Study	Experimental Events	Experimental Time	Control Events	Control Time	IRR	95%-CI
Goldstein et al. (2022)	47	203.00	45	203.00	1.04	[0.69; 1.57]
Taggart et al. (2022)	30	276.00	24	270.00	1.22	[0.71; 2.09]
Taggart et al. (2018)	9	94.50	8	130.50	1.55	[0.60; 4.03]
**Common effect model**					**1.15**	**[0.84; 1.56]**
**Random effects model**					**1.15**	**[0.84; 1.56]**

Heterogeneity: $I^2 = 0\%$, $\tau^2 = 0$, $p = 0.72$

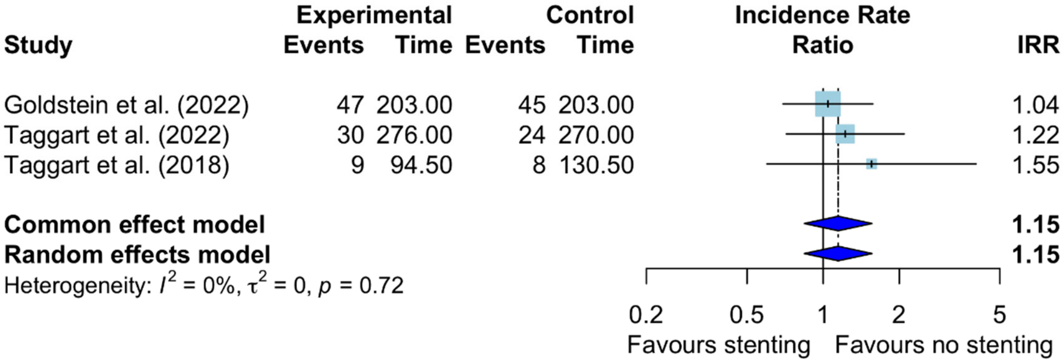

* Taggart et al. (2018). CI: confidence interval; IRR: incidence rate ratio; VEST: venous external support trial.

Supplemental Figure 3. Forest plot for Fitzgibbon patency class I

Study	Experimental Events	Experimental Time	Control Events	Control Time	IRR	95%-CI
Goldstein et al. (2022)	91	203	84	203	1.08	[0.81; 1.46]
Taggart et al. (2022)	72	276	61	270	1.15	[0.82; 1.62]
**Common effect model**					**1.11**	**[0.89; 1.39]**
**Random effects model**					**1.11**	**[0.89; 1.39]**

Heterogeneity: $I^2 = 0\%$, $\tau^2 = 0$, $p = 0.78$

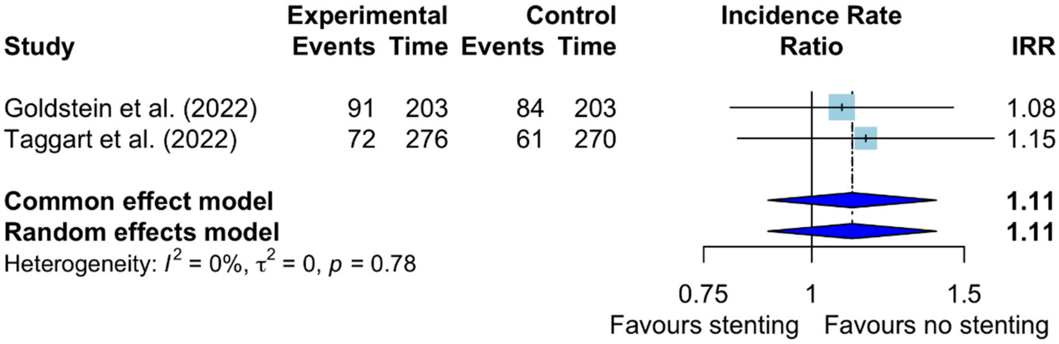

CI: confidence interval; IRR: incidence rate ratio.

Supplemental Figure 4. Forest plot for Fitzgibbon patency classes II-III

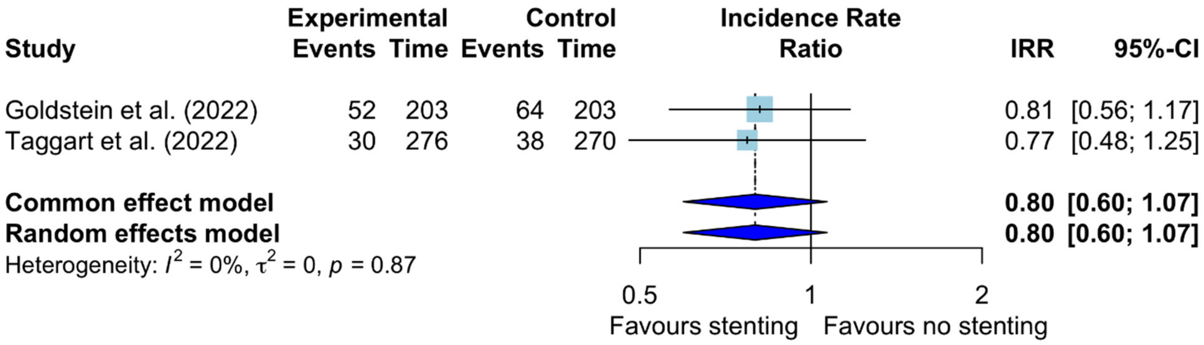

CI: confidence interval; IRR: incidence rate ratio.

Supplemental Figure 5. Forest plot for intimal hyperplasia area

Study	Experimental Total	Experimental Mean	Experimental SD	Control Total	Control Mean	Control SD	SMD	95%-CI
Goldstein et al. (2022)	143	5.11	1.9400	142	5.79	2.4200	-0.31	[-0.54; -0.08]
Taggart et al. (2022)	50	3.07	1.3300	46	3.96	1.3100	-0.67	[-1.08; -0.26]
**Common effect model**	**193**			**188**			**-0.40**	**[-0.60; -0.19]**
**Random effects model**							**-0.45**	**[-0.79; -0.10]**

Heterogeneity: $I^2 = 55\%$, $\tau^2 = 0.0354$, $p = 0.14$

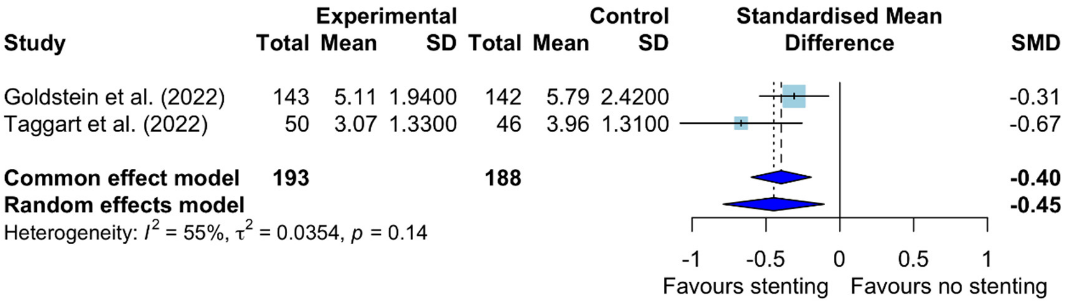

CI: confidence interval; SD: standard deviation; SMD: standardized mean difference.

Supplemental Figure 6. Forest plot for intimal-medial thickness

Study	Experimental Total	Experimental Mean	Experimental SD	Control Total	Control Mean	Control SD	SMD	95%-CI
Goldstein et al. (2022)	143	0.38	0.1400	142	0.43	0.1600	-0.33	[-0.57; -0.10]
Taggart et al. (2022)	50	0.26	0.1080	46	0.34	0.1040	-0.75	[-1.16; -0.33]
**Common effect model**	**193**			**188**			**-0.43**	**[-0.64; -0.23]**
**Random effects model**							**-0.50**	**[-0.90; -0.10]**

Heterogeneity: $I^2 = 66\%$, $\tau^2 = 0.0571$, $p = 0.09$

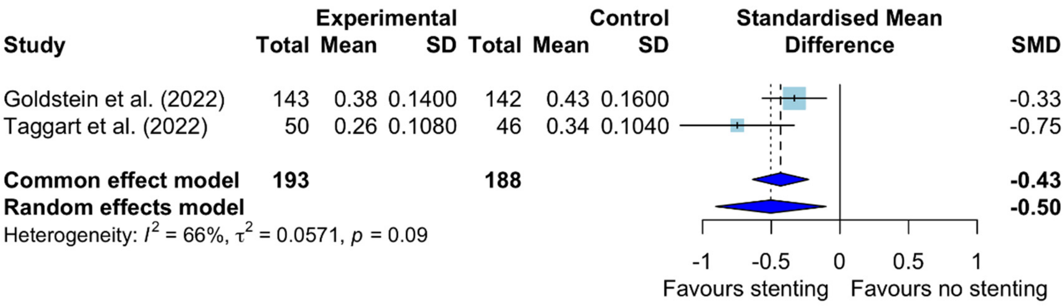

CI: confidence interval; SD: standard deviation; SMD: standardized mean difference.
